# Stress Relaxation Behavior of Azido Propellant Based on BAMO-THF at High Temperatures

**DOI:** 10.3390/ma18010019

**Published:** 2024-12-24

**Authors:** Yiwen Hu, Xiuduo Song, Weilu Yang, Jian Kang

**Affiliations:** 1 Xi’an Modern Chemistry Research Institute, Xi’an 710065, China; 2 State Key Laboratory of Polymer Materials Engineering, Polymer Research Institute of Sichuan University, Chengdu 610065, China

**Keywords:** azido polyether propellant, stress relaxation, mechanical properties, BAMO-THF

## Abstract

The azido propellant, with high energy and low signature, has been a hotpot in the field of propellants. However, the risk of low heat resistance and mechanical performance restricts their range of applications in high-energy formulations. In this study, four azido propellants based on 3,3-bis (azidomethyl) oxetane-tetrahydrofuran copolyether (BAMO-THF) have been prepared, their basic physical properties including energetic properties, internal micro-structure and true density were studied; their tensile properties, dynamic mechanical performances, were investigated, the structure-properties relationship was proposed. The results demonstrate that the obtained propellant shows an elastomeric composite material behavior, with an obvious relaxation in the initial stage and susceptibility to loading condition. The formula structure not only causes obvious difference in the second stage of relaxation, but also strongly affects the initial stage, which is quite different from the influence of testing condition. Besides, the low temperature toughness of the azido propellant is improved by using diol partly replaces diamine as a chain extender, but their stress modulus drop down obviously, leading to the notable stress relaxation behavior at high temperatures. It was found that the improvement of the ordering degree of microstructure or network integrity could restrict the stress relaxation, which was an effective approach to improve the heat resistance and mechanical performance of azido propellant at high temperatures.

## 1. Introduction

In recent years, the azido propellant with 3,3-bis (azidomethyl) oxetane-tetrahydrofuran copolyether (BAMO-THF) as binder, with the characteristics of high energy and low signature, has been widely applied in modern weapons and ammunition [1,2,3,4,5]. However, compared with the traditional hydroxyterminated polybutadiene (HTPB) propellants, BAMO-THF is still not suitable for some practical applications as reported by previous studies [6,7,8], due to its low chain flexibility and poor mechanical properties. Specifically, it is faced with the risk of viscoelastic failure due to softness at high temperatures, which may lead to reduced structural stability [9,10,11]. The risk of low heat resistance and shape instability greatly limits the application depth and breadth of azido propellant in modern weapons [12,13]. Thus, the investigation of mechanical response at high temperatures is crucial for azido propellant based on BAMO-THF.

The relaxation test could provide insights into the stress response of propellant under constant strain, corresponding to the mechanical response of propellant subjected to external deformation during storage, transportation, and flight, which is the result of rearrangement of matrix macromolecular chain under external loading stress [14]. Researchers have studied the relaxation characteristics of propellants by various methods in recent years [15,16,17,18,19,20]. Walid et al. [18] carried out different temperature stress relaxation tests on HTPB propellant by universal testing machine and constructed the master curve of relaxation modulus. They carried out a comparative assessment of three different methods (Williams-Landel-Ferry (WLF) method, the Arrhenius method, and the basic time-temperature superposition (TTS) method) used for estimating and generating the relaxation modulus master curves of ammonium perchlorate-hydroxyl-terminated polybutadiene (AP-HTPB) solid propellant. They suggested that both the WLF and Arrhenius methods can produce satisfactory results when appropriate constants are used, and the WLF method has proven to be more accurate and would be preferred in finite element analysis of AP-HTPB solid propellant. Miller et al. [19] developed a method to determine the stress relaxation master curve of propellants by dynamic mechanical analyzer (DMA). The propellant was tested in uniaxial tension using a conventional approach, and was also tested in a dynamic mechanical analyzer using a dual cantilever beam mode. While the results were similar, the dynamic mechanical analyzer required less material, resulted in reduced variability, and was not sensitive to the applied strain. The quantity of material required was on the order of grams, so that results were obtained with small amounts of propellant, as compared to the conventional uniaxial tension test that requires material quantities on the order of kilograms. Some important considerations also discussed include the verification of strain independence before testing and the loosening of clamps between temperatures during testing. In comparison with the traditional tensile testing method, less time, effort, and materials were required to obtain results. Bihari et al. [21] carried out DMA measurement on six different types of propellants based on hydroxyl terminated polybutadiene, aluminum, and ammonium perchlorate cured with toluene diisocyanate having burning rates varying from 5 mm/s to 25 mm/s at 7000 kPa. Each propellant sample was given a multi-frequency strain of 0.01 percent at three discrete frequencies (3.5 Hz, 11 Hz, 35 Hz) in the temperature range of −80 °C to +80 °C. All the propellants were found to exhibit two relaxation events (α- and β-transition) in the temperature range of −80 °C to +80 °C. The activation energy for both transitions was determined by Arrhenius plot from dynamic properties measured at different frequencies, and was also determined by time temperature superposition principle using WLF and Arrhenius temperature dependence equations.

For the mechanical response of the azido propellant under the coupling of temperature and external load, the new testing instrument, analysis method, and simulation technology were employed to provide further information for the optimization of mechanical properties [22,23,24,25]. Zheng et al. [26] studied the impact loading simulation experiment of azide polyether propellant at −40 °C using an instrument-based drop hammer impact testing machine. The impact damage characteristics of the propellant were characterized by X-ray micro tomography (X-μCT), and the dynamic mechanical properties of azide polyether propellant at different loading frequencies were studied using DMA. They reported that the dynamic mechanical parameters of the propellant exhibited significant loading frequency dependence. When the loading frequency increased from 1 Hz to 20 Hz, its glass transition temperature increased from −38.1 °C to −23.1 °C. Under the corresponding testing conditions, the propellant sample fractures when the impact loading energy is not less than 2 J. The propellant without fracture does not produce macroscopic cracks, but the ammonium perchlorate (AP) particles inside have been partially broken. The impact curve of the fractured sample contains an unstable crack propagation process, indicating that the propellant has certain brittle material characteristics at low temperatures. It is speculated that the damage mode is that the AP particles containing defects first undergo transgranular fracture, and the initial microcracks propagate in the matrix and interpenetrate, ultimately leading to the fracture of the sample. Hu [13] also studied the impact damage of azido polyether propellant by the instrumented drop hammer device at −40 °C, and characterized the impact damage by X-ray computed microtomography (X-μCT). Their studies revealed the impact damage mechanism of azido polyether propellant under impact loading and low temperatures, promoting the study of damage mechanics of high-energy propellant. However, responding to guide the engineering application, the basic research on the mechanical behavior, and its influencing factors of BAMO-THF based azido propellant is insufficient.

In this paper, the DMA method was used to investigate the stress relaxation of BAMO-THF based azido propellant under high temperatures. The mechanical response characteristics of the propellant under different conditions (different strain levels and temperatures) and different formulations are discussed in detail. Moreover, the influence of different related factors on the relaxation process was also analyzed.

## 2. Experimental Section

### 2.1. Materials

BAMO-THF with *M*_n_ = 2770 g mol^−1^, butyl nitroxyethylnitramine (BuNENA), and A3 (mixture of bis-(2,2-dinitropropyl)-acetal and bis-(2,2-dinitropropyl) formal with the mass ratio of 1:1, BDNPA/BDNPF) were provided by Liming Research Institute of Chemical Industry (Luoyang, China). The trimethylolpropane (TMP, as crosslinking agent), 2,4-toluenediisocyanate (TDI, as curing agent), 3,3′-dichloro-4,4′-diaminodiphenylmethane (MOCA) or 1,4-butanediol (BDO) as chain extender were purchased from Sigma-Aldrich Fine Chemicals, Darmstadt, Germany. Other reagents were all analytical grade and used as received.

### 2.2. Sample Preparation

BAMO-THF based azido propellant was prepared by slurry casting process according to the manner proposed in literature [26]. The main parameters are listed in Table 1, where *R* is the curing parameter, *C*_D_ is the chain extension parameter, *C*_T_ is the crosslinking parameter, and *p*l/*p*o is the mass ratio of plasticizer and polymer.

**Figure 1:**
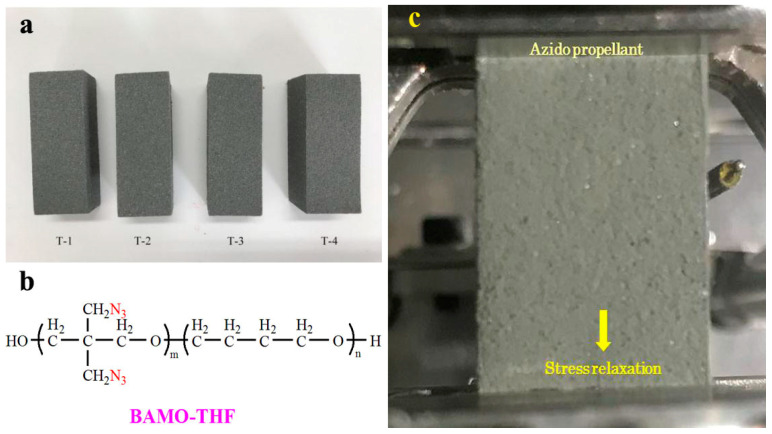
The azido propellants based on BAMO-THF: (**a**) macrograph, (**b**) molecular structure of BAMO-THF, and (**c**) test process of stress relaxation.

The curing parameters, crosslinking parameters and chain extension parameters are the molar ratio of curing agent, crosslinking agent, and chain extender functional groups to the total functional groups, respectively. The prepared propellants are shown in Figure 1a.

### 2.3. Experimental Setup

The energetic properties of the azido propellants were calculated by means of the “Energy Calculation Star (ECS)” software version 1.0, which was developed by Xi’an Modern Chemistry Research Institute based on the fundamental thermodynamic principle of minimum free energy [27]. It is mainly used to calculate the energy characteristics of solid propellants and gunpowder force of propellants, as well as to determine the equilibrium characteristics, chemical composition, and phase composition of any chemical system under given conditions. It can also be used to calculate thermodynamic and thermophysical properties. The initial assumption for theoretical calculation is that the combustion chamber pressure is 7 MPa, the nozzle outlet pressure level is 0.1 MPa, and the chemical formula and heat generation data of the formula components are identical. The parameters in Table 1 were used for calculation.

**Figure 2:**
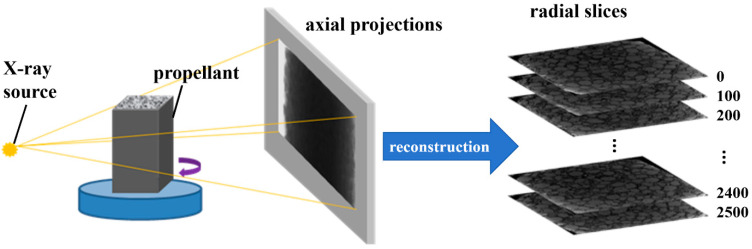
Schematic diagram of X-μCT scanning.

The internal microstructure of BAMO-THF based azido propellant was recorded by X-μCT (Scanco μCT80, Zurich, Switzerland. Pixel resolution: 0.5 μm; realistic low contrast 3D resolution: 2 μm; testing principle seen in Figure 2).

The measurement was performed according to previous study [28].

The true density of each azido propellant was characterized by density analyzer (Micrometrics Accupyc 1340, Micromeritics Instrument Corporation, Norcross, GA, USA), with a testing accuracy of ±0.01%. For each analysis, a 0.152 g sample was weighed directly into the cup. The cup containing the sample was placed inside the cell, which thereafter was placed inside the sample compartment and sealed. Nitrogen was passed into the sample compartment until it reached an equilibrium rate of 0.0050 psig/min. After pressure stabilization, gas was allowed to expand into the reference compartment. The pressure before and after expansion was measured automatically and used to compute the sample volume. The ratio of known sample mass and volume yields the density.

The tensile properties of the samples were investigated by uniaxial tensile tester (INSTRON 3400, INSTRON Corporation, Norwood, MA, USA, maximum load: 10 kN–100 kN, test force measurement range: 5~50 kN, equipped with low/high temperature chamber). Five replicate specimens with the size of 5 mm × 10 mm × 30 mm were taken for each test, and the average data were reported. Tensile test was performed with the speed of 100 mm min^−1^ at different temperatures (−40, 20, and 50 °C) respectively.

The glass transition temperature (*T*_g_), strain rate dependency, and temperature dependency of dynamic mechanical performance of the propellant were analyzed by Q2000 differential scanning calorimeter (DSC, TA Instruments, New Castle, DE, USA) with the calorimetric accuracy of ±0.05 °C, sensitivity of 0.2 microwatts, and relative resolution of 2.9. For the measurement of *T*_g_, a sample was tested using a stretching bracket, with the testing condition of standard mode, 60 μm amplitude, 0.5 Hz frequency, 0.5 N static force, 15 N dynamic force, 1.1 scaling factor, and heating range of −80 °C to 50 °C at 5 °C/min. For strain rate dependency measurement, the test was performed using a stretching bracket and the strain levels of 0.5%, 1%, and 2% at 25 °C were selected. For temperature dependency measurement, the test was carried out using a stretching bracket, at 30, 40, and 50 °C respectively, and the strain level was 0.5%.

The relaxation mechanical properties of the propellant were studied by dynamic mechanical analyzer (DMA-Q800, TA Instruments, New Castle, DE, USA, shown in Figure 1c) with the modulus accuracy of ±1%, tan δ resolution of 0.00001, and constant temperature stability of ±0.1 °C, operated in single-frequency strain mode using a single-cantilever clamp. The samples were balanced for 5 min before the test, and then relaxed for 45 min under the corresponding test conditions.

## 3. Results and Discussion

### 3.1. Basic Physical Properties of the Propellants

#### 3.1.1. Theoretical Energetic Performance Calculation of the Propellants

The energetic performance of the obtained propellants is shown in Table 2. The prepared azido propellants have high energy levels, with the theoretical specific impulse of approximately 268 s and average density of 1.79 g cm^−3^. Since the HMX has a higher enthalpy of formation compared with AP, the increase of HMX fraction results in further improvement of energy level in the T-2. When the proportion of matrix in propellant formula is constant, the increase of the plasticizing ratio in T-4 formula is equivalent to that, and the nitrate plasticizer A3 partially replaces BAMO-THF elastomer, leading to the slight improvement of theoretical specific impulse.

#### 3.1.2. Internal Morphology of the Propellants

**Figure 3:**
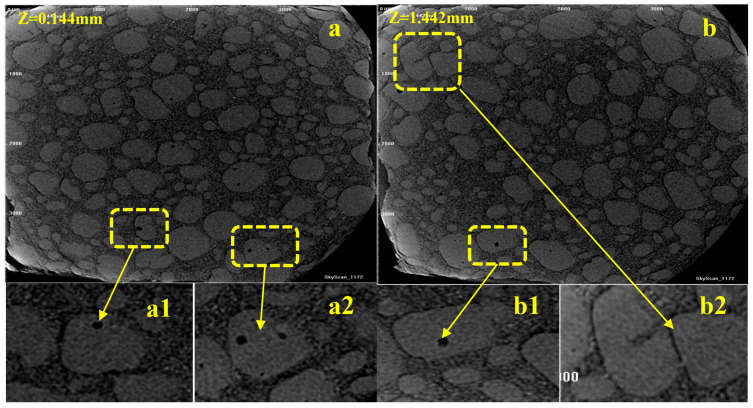
The internal micrograph by X-μCT: (**a**) scanning depth Z = 0.144 mm, and (**b**) scanning depth Z = 1.442 mm.

To investigate the internal micromorphology of BAMO-THF based azido propellants, X-μCT was employed to provide further insights into the detailed information (shown in Figure 3).

The dark gray area illustrated above is mainly adhesive matrix, the bigger particle of light gray area is AP (with the particle size of ~100 μm) [11], and the smaller one is HMX (with the particle size of ~26 μm) [13]. The prepared propellant has a relatively dense and uniform structure. In addition, the crystals of AP and HMX with a regular shape are well-dispersed in the continuous phase of BAMO-THF matrix. Meanwhile, some defects such as voids and interfacial debonding of the binder from the larger AP particles can be found in a few AP particles (a1, a2, b1, and b2 area in Figure 3). The little interfacial debonding might be induced by the difference of thermal expansion coefficient between propellant components during the curing process [26,27].

#### 3.1.3. Mechanical Performance of the Propellants

As an energetic material for practical application scenarios, the propellants are expected to have robust mechanical properties at various operating temperatures. Therefore, we selected three representative temperatures in actual working conditions (−40, 20 and 50 °C) and tested the mechanical properties of the propellants.

**Figure 4:**
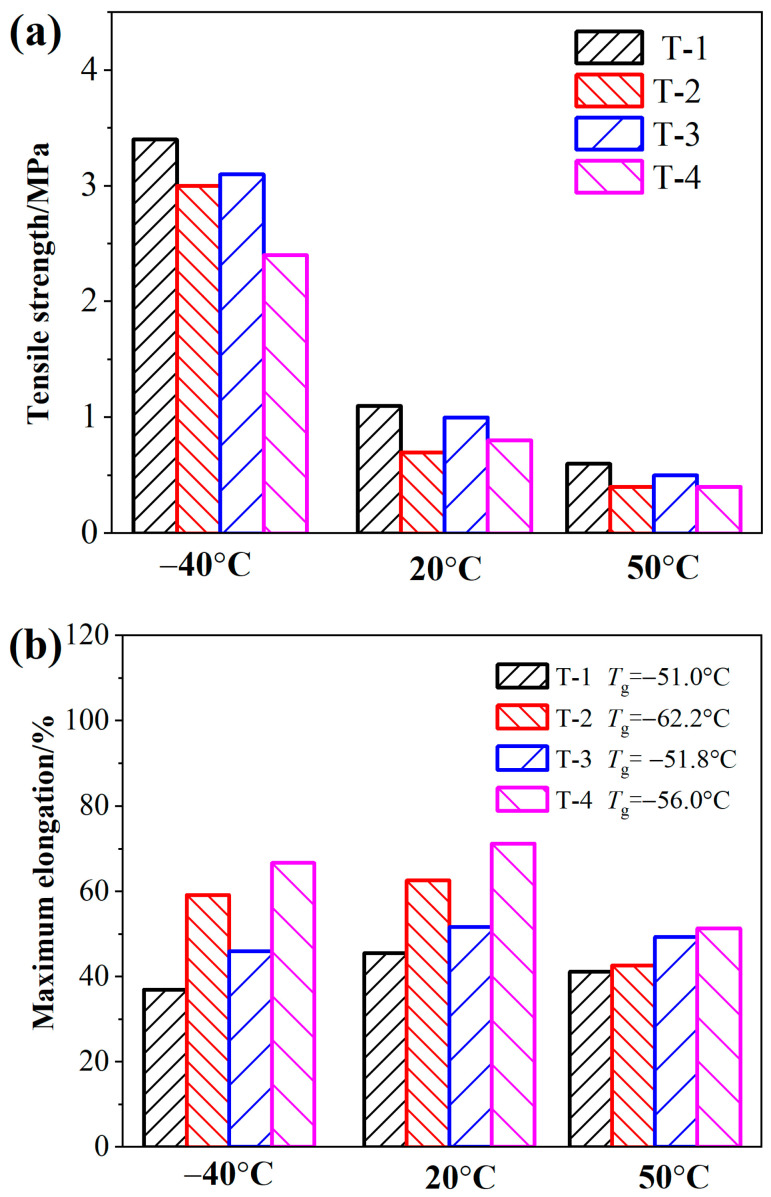
The tensile strength (**a**) maximum elongation and glass temperature *T*_g_, and (**b**) of azido propellants based on BAMO-THF measured at different temperatures.

The basic mechanical properties of BAMO-THF based azido propellants are shown in Figure 4.

Significant reduction of *T*_g_ value and evident increase of maximum elongation in T-2 is observed, suggesting that the viscoelasticity of the propellants is greatly improved with the application of BuNENA. In other words, BuNENA has a better plasticizing ability than A3 as an energetic plasticizer [29]. Meanwhile, the tensile strength is partially weakened. Similarly, when the plasticizing level is increased from 1.0 to 1.2, and elongation and partial reduction of strength is greatly improved, as seen in Figure 4 (T-3 and T-4). These results suggest that the increase of plasticizing level or content of plasticizer not only significantly improves the ductility of azido propellants, but also reduces mechanical strength [24]. This phenomenon could be attributed to the intermolecular lubrication of plasticizer to the polymer molecular chain. The promotion of plasticizing level can reduce the intermolecular force of the matrix, leading to the easier deformation under external force [25]. Furthermore, it can be also found that the diol chain extenders have partially replaced the diamine chain extenders in T-1 and T-3. The tensile strength changes little at low temperature, but the maximum elongation increases significantly. These results suggest that the combination of diamine/diol chain extenders can improve the low temperature toughness of azido propellants based on BAMO-THF.

### 3.2. Stress Relaxation Behavior of the Propellants

#### 3.2.1. Strain Rate Dependence of the Propellant

**Figure 5:**
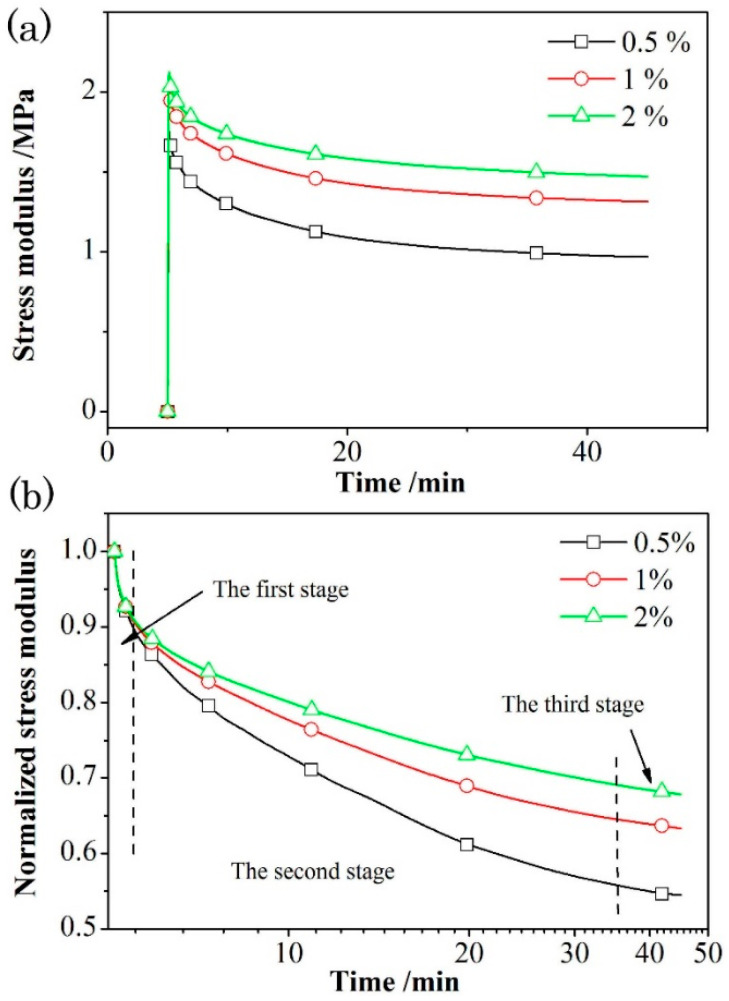
Relaxation modulus (**a**) and normalized relaxation modulus (**b**) of azido propellants T-1 under different strain.

The initial strain has a great influence on the structural change of propellant during stress relaxation. In this section, the strain rate dependence of T-1 was studied as a representative, since the results of the four propellants show similar trends. The external forced deformation level of grain was small in the process of propellant grain storage, transportation, ignition, and flight. The testing strain levels of 0.5%, 1%, and 2% were selected to investigate the influence of forced deformation on the relaxation modulus of the propellants T-1 as a representative. Figure 5 shows the relaxation modulus curves and normalized relaxation modulus curves. The normalized relaxation modulus is the ratio of the measured modulus to the initial maximum modulus.

It can be observed from Figure 5a that after 40 min, the slope of each relaxation curve is close to 0, indicating that the stress relaxation behavior of the propellant sample basically reaches an equilibrium state within the testing time range. The relaxation modulus of propellant with strain values of 0.5%, 1%, and 2% at 50 °C are 1.78 MPa, 2.02 MPa, and 2.17 MPa, respectively. The effect of strain level on the relaxation modulus is obvious, which could be attributed to the forced deformation through the movement of molecular chain under external loading stress [30]. Moreover, in a small deformation range, the increase of the forced deformation strain leads to the enhancement of stress modulus, which is attributed to the rapid improvement of the molecular chain movement barrier of polymer matrix.

The normalized relaxation modulus curves can help to provide better understanding in the effect of strain levels on the stress relaxation behavior of BAMO-THF based azido propellants. Figure 5b shows that the normalized stress curve can be divided into three stages in general. The first stage is mainly induced by elastic relaxation, where the normalized curves of relaxation modulus at different strains are almost identical, indicating that the samples under different strain levels have almost the same initial stress relaxation rate [14]. This phenomenon indicates that the rate of initial stress relaxation in propellant has little correlation with the strain level. In addition, the third stage could be attributed to the viscous relaxation [16,17], where the slope of the normalized curve at different strains are basically the same, demonstrating that the strain level has little effect on the relaxation rate at the late stage of stress relaxation. In contrast, the difference of stress relaxation caused by different strain levels is mainly reflected in the second stage, which is the result of the joint contribution of viscosity and elasticity [14,17,31]. It can be found that the attenuation of relaxation modulus is opposite to the tendency of stain level. In other words, the smaller strain level exhibits more obvious attenuation of relaxation modulus. This phenomenon could be attributed to the difference of the joint contribution of viscosity and elasticity [31].

#### 3.2.2. Temperature Dependence of the Propellant

**Figure 6:**
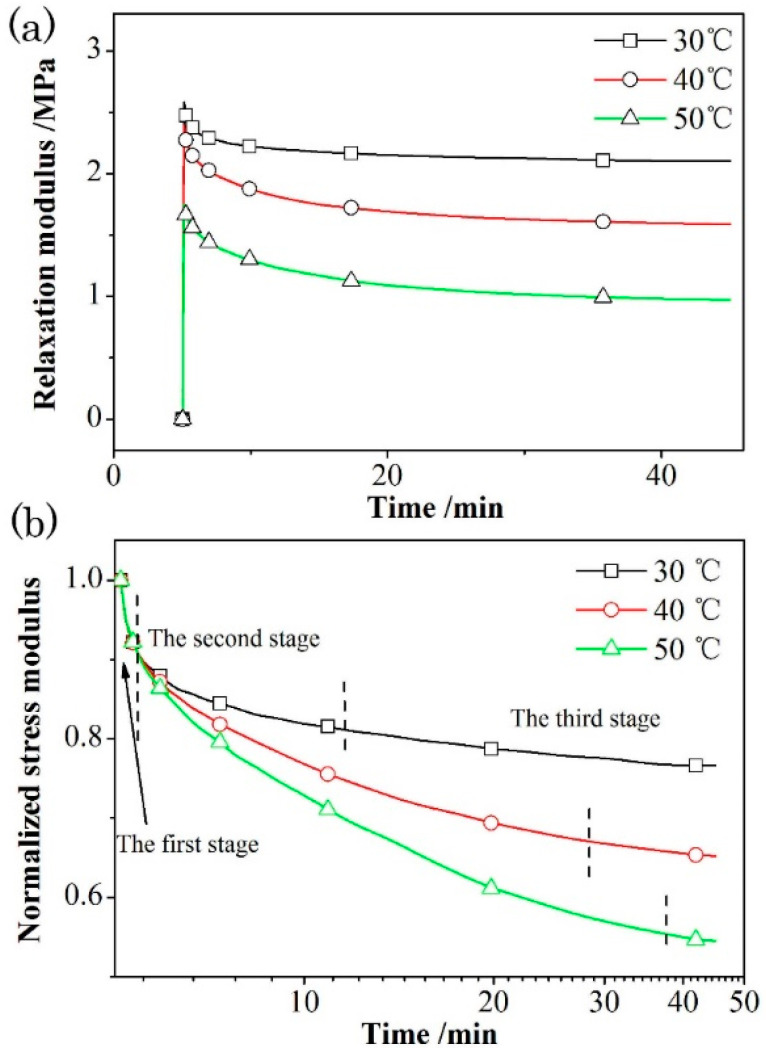
Relaxation modulus (**a**) and normalized relaxation modulus (**b**) of azido propellants T-1 under different temperatures.

Figure 6 represents the results of stress relaxation modulus and normalized relaxation modulus of propellants T-1 as a representative at different temperatures with 0.5% strain level, since the results of the four propellants show similar trends.

A gradual decrease in the relaxation modulus can be seen in Figure 6a as the testing temperature increases, indicating that the viscous part of BAMO-THF matrix is more obvious at high temperatures. With the increase of temperature, the free volume of BAMO-THF macromolecular segment increases, and the chain segments are easy to move under the external loading stress, resulting in the decrease in relaxation modulus [32]. Besides, there are differences in the three stages of normalized stress relaxation curves measured at different temperatures (Figure 6b). The second stage is greatly affected by the test temperature, and the relaxation time increases with the increase of temperature [29]. Moreover, the relaxation rate also depends on the test temperature, which increases with the increase of the test temperature. This phenomenon is quite different from the first stage (elastic stage), where the temperature has little influence on the relaxation rate, with almost identical relaxation normalization curves at different temperatures. In the third stage (viscous stage), the slopes of the normalization curves are close to each other, and the relaxation rate is small, and the influence of temperature on the relaxation rate of propellant in the later stage of stress relaxation is very small. Therefore, it can be inferred that the effect of temperature on the relaxation process of propellant is significant only in the second stage. In general, the stress relaxation trends of different temperature levels are basically the same, where the stress relaxation is obvious in the initial stage of the relaxation process, and the relaxation is slowed down over time.

#### 3.2.3. Formulation Dependence of the Propellants

**Figure 7:**
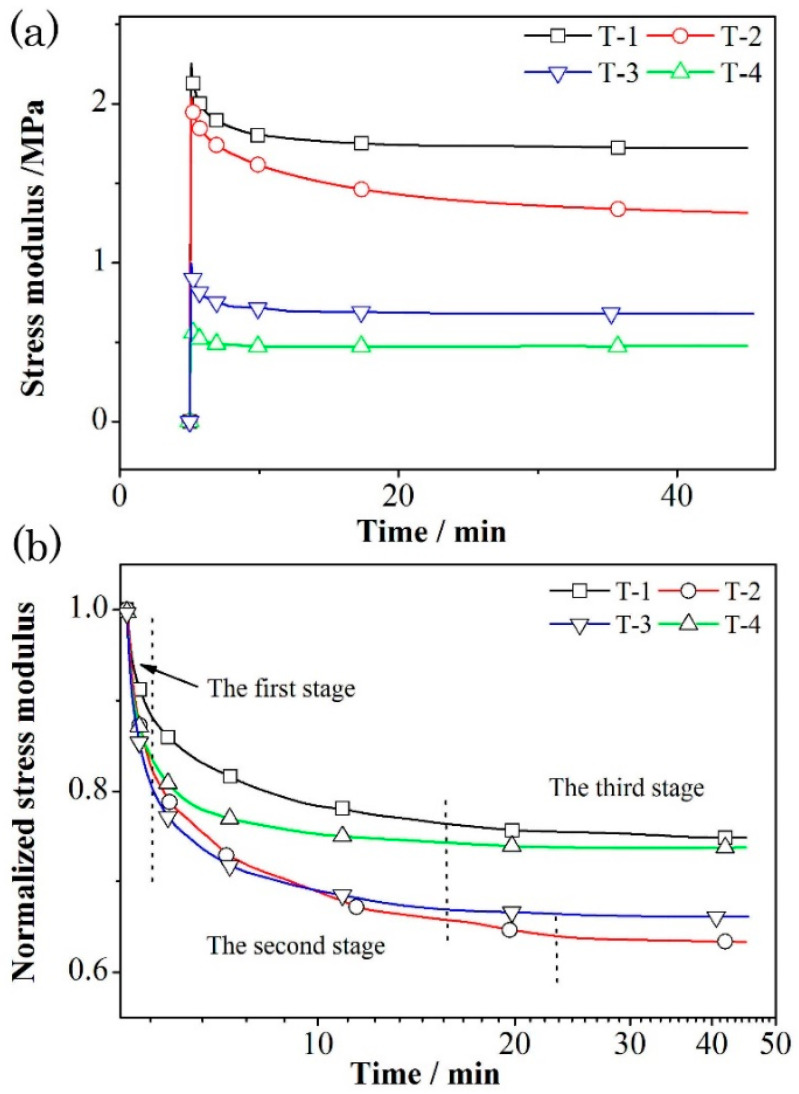
Relaxation modulus curves (**a**) and normalized relaxation modulus curves (**b**) of azido propellants based on BAMO-THF under different formulas.

The effect of different formulations on the stress relaxation behavior of azido propellant is further studied. Figure 7 shows the curve of relaxation modulus with time under the test temperature of 50 °C and strain of 0.5%.

**Figure 8:**
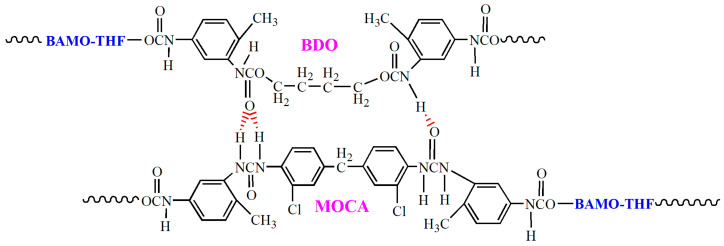
The illustration of hydrogen bonding in binder matrix based on BAMO-THF.

It can be seen from Figure 7a that the relaxation modulus for propellant formulas T-1 and T-2 are significantly higher than those of formulas T-3 and T-4, which are mainly caused by the difference in physical network structure. This is consistent with the viscoelastic law observed in the high temperature creep process of propellants [33]. After curing, the amino group (-NH-) in amine chain extender (MOCA) is prone to form hydrogen bonding, resulting in microphase separation in the adhesive matrix, where the microstructure of the hard segment is highly ordered (as shown in Figure 8) [5,34]. Thus, the mechanical reinforcement effect of MOCA on BAMO-THF adhesive matrix is more obvious than that of alcohol chain extender (BDO), resulting in the higher relaxation modulus of T-1 and T-2 formulas. Furthermore, it can also be seen in Figure 4a that the relaxation modulus of formula T-1 is higher than that of formula T-2, and that of formula T-3 is higher than that of formula T-4, which is consistent with the results from tensile test. The small molecular plasticizers can improve the viscosity of propellant, reduce the “internal friction” between polymer molecular chains, and make the molecular chains to move more easily under the same external conditions, leading to the smaller stress response value.

The stress relaxation process of propellant can be explained by the conformation change of BAMO-THF random co-polyether matrix. Before the relaxation experiment, the molecular chains in BAMO-THF matrix network structure are curled and intertwined [17,33]. When subjected to deformation load, some molecular chains will be straightened to some extent, but they are still entangled with each other. The “stretched” molecular chains will bear and counteract the external stress. With the extension of time, the molecular chains of the BAMO-THF move through the chain segment, slowly adjust the conformation, and return to a relatively free and stable state (curl state), resulting in the gradual attenuation of stress response value. However, the BAMO-THF matrix is a cross-linked network structure, which cannot experience flow deformation under external force, and certain stress is needed for the matrix to maintain an elastic deformation in the stretching process. Therefore, the relaxation modulus of the propellant tends to be a stable value (Figure 7a).

In the stress relaxation normalization curves (Figure 7b), the change of formula structure causes obvious difference in the elastic relaxation stage of the propellants, which is quite different from the effect of test conditions (strain level and test temperature). This could be because only the elastic part, such as the bond angle and bond length, will change in this process when stress is applied [9,32]. Therefore, the improvement of ordering degree of the matrix or network integrity can effectively inhibit the mutual sliding of the molecular chains, leading to the difference in the relaxation rate. In the second stage, the movement of BAMO-THF polymer segments starts to be excited with a short-range diffusion movement, where the molecular chain changes from the curly state to the elongation state [9]. Thus, an obvious relaxation process could be observed. The factors that affect the molecular chain movement of matrix, such as matrix chemistry and physical network structure, plasticizer, etc., will affect the relaxation process. Hence, the relaxation process of the four propellant formulations in the second stage is quite different. The T-2 formula with BuNENA has a higher relaxation rate and a longer relaxation time, resulting in a higher relaxation ratio than the other three formulations.

## 4. Conclusions

In this study, four azido propellant based on BAMO-THF were prepared. Following this, their basic physical properties including energetic properties, internal microstructure, and true density were studied. We found that these azido propellants have relatively dense and uniform structure, with the theoretical specific impulse of around 268 s and average density of 1.79 g cm^−3^. The propellants show a typical polyurethane composites behavior, with an obvious relaxation in the initial stage and attenuation over time. The tensile properties and dynamic mechanical performances of the propellants were investigated. After conducting the tensile tests at −40, 20, and 50 °C, we found that the increases of plasticizing level or plasticizer content not only significantly improve the ductility of azido propellants, but also cause the reduction of mechanical strength. DMA suggested that the influence of the loading condition on the relaxation process takes place in the second stage. Furthermore, the combination of diamine/diol chain extenders can improve the low temperature toughness, but their stress modulus drops down obviously, leading to significant relaxation behavior. Compared with the chain extenders, the influence of the increase of plasticizing level or plasticizer content is not obvious. Moreover, we found that the change of formula structure not only causes obvious difference in the second stage, but also strongly affects the first stage, which is quite different from the effect of testing condition. The improvement of the ordering degree of the microstructure or the network integrity can effectively inhibit the stress relaxation process. The results of this manuscript provided new understanding concerning the structure-properties relationship, especially the stress relaxation behavior of azido propellant based on BAMO-THF at high temperatures. Moreover, the characterization method proposed in this study is useful for related studies.

## Figures and Tables

**Table 1:** The composition of azido propellants based on BAMO-THF.

Formulations	AP/HMX/Al	*R*	*C*_D_	*C*_T_	Plasticizers	*pl*/*po*	Chain Extenders
T–1	45/12/18	1.06	0.8	0.25	A3	1.0	MOCA
T–2	42/15/18	1.06	0.8	0.25	A3/BuNENA	1.0	MOCA
T–3	45/12/18	1.06	0.8	0.25	A3	1.0	MOCA/BDO
T–4	45/12/18	1.06	0.8	0.25	A3	1.2	MOCA/BDO

**Table 2:** The energetic performance of azido propellants.

Formulations	Density (g cm^−3^)	Theoretical Specific Impulse (s)	Heat of Explosion (kJ kg^−1^)	Theoretical Flame Temperature (K)
T-1	1.79	268.2	5659	3684
T-2	1.79	269.8	5606	3645
T-3	1.79	268.2	5659	3684
T-4	1.79	268.0	5654	3681

## Data Availability

The original contributions presented in the study are included in the article. Further inquiries can be directed to the corresponding author.
